# Reply to Ocklenburg and Mundorf: The interplay of developmental bias and natural selection

**DOI:** 10.1073/pnas.2205299119

**Published:** 2022-07-05

**Authors:** Iain G. Johnston, Kamaludin Dingle, Sam F. Greenbury, Chico Q. Camargo, Jonathan P. K. Doye, Sebastian E. Ahnert, Ard A. Louis

**Affiliations:** ^a^Department of Mathematics, University of Bergen, Bergen 5007, Norway;; ^b^Computational Biology Unit, University of Bergen, Bergen 5008, Norway;; ^c^Rudolf Peierls Centre for Theoretical Physics, University of Oxford, Oxford OX1 3PU, United Kingdom;; ^d^The Alan Turing Institute, British Library, London NW1 2DB, United Kingdom;; ^e^Centre for Applied Mathematics and Bioinformatics, Department of Mathematics and Natural Sciences, Gulf University for Science and Technology, Hallwaly, Kuwait;; ^f^Theory of Condensed Matter Group, Cavendish Laboratory, University of Cambridge, Cambridge CB3 0HE, United Kingdom;; ^g^Department of Metabolism, Digestion and Reproduction, Imperial College London, London SW7 2AZ, United Kingdom;; ^h^Department of Computer Science, University of Exeter, Exeter EX4 4QF, United Kingdom;; ^i^Physical and Theoretical Chemistry Laboratory, Department of Chemistry, University of Oxford, Oxford OX1 3QZ, United Kingdom;; ^j^Department of Chemical Engineering and Biotechnology, University of Cambridge, Cambridge CB3 0AS, United Kingdom

In our paper (1), we argue for strong bias in the arrival of variation toward phenotypes with simple descriptions. Evaluating evidence for such hypotheses about developmental bias is hard because one needs to answer counterfactual questions (2, 3). Not only “What do we observe in nature?” but also “What could have happened, but did not occur?” We therefore focused on relatively simple systems where such questions are potentially tractable. For protein complexes, this bias translates into a hugely enhanced probability of obtaining symmetric structures. For RNA structure and a gene regulatory network, the pattern of simpler outcomes is similarly pronounced, but has a less evocative interpretation.

The interesting comment of Ocklenburg and Mundorf (4) provides an opportunity to discuss the big question of whether such developmental bias also carries through for evolution at larger lengths-scales. A good place to start may be the classic examples of large-scale structures that are generated by relatively simple algorithmic processes such as the fractal structure of lungs and vasculature, the shapes of plants (5), and, potentially, brain structure. While the morphological patterns observed on these scales are algorithmically simple, they are not necessarily symmetric (symmetry being a special case of the more general bias toward simplicity).

The second big question, which lies at the heart of the comment (4), is whether nervous systems and brain architecture are examples where asymmetry has a functional advantage in biology. We see no reason to disagree. Indeed, complex/asymmetric structures abound across biological scales, from molecular machines, through cellular and tissue structures, to organismal body plans. In many cases, functional advantages of asymmetry can be determined. Even protein clusters show small deviations from perfect symmetry, in part because perfect symmetry creates unnatural chemical bonds and angles at the interfaces between the units (6, 7). Positive and negative adaptive pressure away from perfect symmetry may also hold for brains. Interestingly, functional hemispheric asymmetries are much stronger than anatomical asymmetries, suggesting a complex evolutionary coupling between function and structure (8).

Finally, we predict two measurable consequences of this balancing between a general favoring of simplicity from the algorithmic nature of evolution and specific selective pressures on form. Firstly, earlier evolutionary morphologies should be simpler than later ones, where there has been time to explore a larger set of rarer potential variation that may, in turn, be more adaptive. Secondly, random mutations should lead to simpler structures, and possibly even recapitulate evolutionary histories. We cite examples from mammalian dentition (9) and leaf formation in angiosperms (10) in our discussion section, and we could include others such as protein complexes (11). Could these specific hypothesized effects, or other signatures of the interplay between bias in the arrival of variation and the adaptive pressures of natural selection, be observed in the evolution of brains? Surely such questions rank among the greatest scientific challenges of our time.
